# Genome-Wide DNA Methylation Indicates Silencing of Tumor Suppressor Genes in Uterine Leiomyoma

**DOI:** 10.1371/journal.pone.0033284

**Published:** 2012-03-13

**Authors:** Antonia Navarro, Ping Yin, Diana Monsivais, Simon M. Lin, Pan Du, Jian-Jun Wei, Serdar E. Bulun

**Affiliations:** 1 Division of Reproductive Biology Research, Department of Obstetrics and Gynecology, Feinberg School of Medicine, Northwestern University, Chicago, Illinois, United States of America; 2 Bioinformatics Core, Feinberg School of Medicine, Northwestern University, Chicago, Illinois, United States of America; 3 Department of Pathology, Feinberg School of Medicine, Northwestern University, Chicago, Illinois, United States of America; Virginia Commonwealth University, United States of America

## Abstract

**Background:**

Uterine leiomyomas, or fibroids, represent the most common benign tumor of the female reproductive tract. Fibroids become symptomatic in 30% of all women and up to 70% of African American women of reproductive age. Epigenetic dysregulation of individual genes has been demonstrated in leiomyoma cells; however, the *in vivo* genome-wide distribution of such epigenetic abnormalities remains unknown.

**Principal Findings:**

We characterized and compared genome-wide DNA methylation and mRNA expression profiles in uterine leiomyoma and matched adjacent normal myometrial tissues from 18 African American women. We found 55 genes with differential promoter methylation and concominant differences in mRNA expression in uterine leiomyoma versus normal myometrium. Eighty percent of the identified genes showed an inverse relationship between DNA methylation status and mRNA expression in uterine leiomyoma tissues, and the majority of genes (62%) displayed hypermethylation associated with gene silencing. We selected three genes, the known tumor suppressors KLF11, DLEC1, and KRT19 and verified promoter hypermethylation, mRNA repression and protein expression using bisulfite sequencing, real-time PCR and western blot. Incubation of primary leiomyoma smooth muscle cells with a DNA methyltransferase inhibitor restored KLF11, DLEC1 and KRT19 mRNA levels.

**Conclusions:**

These results suggest a possible functional role of promoter DNA methylation-mediated gene silencing in the pathogenesis of uterine leiomyoma in African American women.

## Introduction

Uterine leiomyomas or fibroids are benign smooth muscle tumors of myometrial origin; despite their benign nature, they are able to undergo rapid and considerable growth [1]. Uterine leiomyomas are the most common gynecological tumors in women of reproductive age, and they become symptomatic in 25–30% of all women and in up to 70% of African American women of reproductive age [2]. Compared with white women, African American women are 3 times more likely to develop symptomatic leiomyoma, which also develops at earlier ages with more numerous and larger fibroids [3]. The clinical symptoms associated with uterine leiomyoma are abnormal uterine bleeding, which can lead to anemia, pelvic pressure and pain; reduced fertility; and frequent pregnancy loss [4]. In the United States, 600,000 hysterectomies are performed each year; of these, approximately 40% are performed to treat uterine leiomyoma. The surgical costs alone represent an economic burden of $2 billion per year [5], and when taking into account the social costs and associated long-term health problems, it is clear that better prevention and treatment options for women with uterine leiomyoma are urgently needed. Understanding the molecular mechanisms underlying the pathogenesis of uterine leiomyoma will facilitate the discovery and development of new approaches to the treatment of this disease.

Gene expression profile studies have demonstrated that hundreds of genes with critical functions in differentiation, apoptosis, proliferation and extracellular matrix formation are dysregulated in uterine leiomyoma [6]. Currently, a few cytogenetic aberrations in specific genes have been discovered [7]; however, it remains unknown whether these dysregulated genes act as effectors or growth promoters in uterine leiomyoma. Epigenetic mechanisms such as DNA methylation, histone modification, and non-coding RNAs are described as heritable changes in gene expression not associated with changes in the primary DNA sequence; rather, these changes affect secondary interactions that play a crucial role in the regulation of gene expression. In the mammalian genome, DNA methylation is the most common and well-characterized epigenetic mark, which consists of the covalent addition of a methyl group to the 5′-carbon of the cytosine ring within the context of CpG dinucleotides following replication. The methylation of this cytosine is catalyzed by specific DNA methyltransferases (DNMTs), which transfer a methyl group, from the donor S-adenosyl methionine (SAMe) to the 5′-position of the pyrimidinic ring [8]. Recent studies reveal that there is differential expression of DNMTs in uterine leiomyoma and that there is aberrant DNA methylation in uterine leiomyoma compared with normal myometrial tissue [9]–[10]. One study demonstrated that hypomethylation of ESR1 in uterine leiomyoma correlates with increased mRNA expression in uterine leiomyoma [11]. These findings suggest that, DNA methylation might play a key role in the pathogenesis of uterine leiomyoma by altering the normal myometrial mRNA expression profile. Further characterization of the role of epigenetics in the tumorigenesis of uterine leiomyoma, requires an analysis of global, genome-wide DNA methylation in disease and normal uterine tissue.

The objective of this study was to determine the relationship between differential DNA methylation and mRNA expression in uterine leiomyoma by performing a genome-wide analysis. We sought to determine whether differentially regulated genes in uterine leiomyoma versus adjacent normal myometrial tissue are under epigenetic control. We attempted to identify a subset of genes whose differential DNA methylation correlated with differential mRNA expression. Our findings will advance our understanding of the contribution of DNA methylation to the pathogenesis of uterine leiomyoma.

## Results

### Analysis of DNA methylation and mRNA expression in uterine leiomyoma and matched adjacent myometrial tissue

We compared DNA methylation patterns in human uterine leiomyoma and adjacent normal myometrial tissues from 18 women to identify genes that are differentially expressed and epigenetically regulated. We used samples from African American women to limit biological heterogeneity and avoid epigenetic variation among ethnic groups; key clinical characteristics of samples are described in Table 1. We performed a genome-wide DNA methylation analysis using the high throughput Illumina Infinium Human Methylation27 Beadchips in paired sets of uterine leiomyoma and matched adjacent normal myometrial tissues. The Illumina probes detect the methylation state of 27,578 individual CpG dinucleotides, located predominantly in CpG islands within proximal promoter regions between 1.5 kb upstream and 1 kb downstream of the transcription start sites of 14,475 consensus coding sequences genes throughout the human genome [12]. To complement this approach, we also performed parallel genome-wide mRNA expression profiling using the Human Ht-12v3 expression beadchip, which targets more than 25,203 transcripts. These two platforms share approximately 66% genes; thus, the majority of the human genes were represented in our genome-wide analysis.

**Table 1:** Descriptive characteristics of subjects.

Subject No	Age	Weight (gm)	Size^1^ (cm)	No of tumors in uterus	Cycle phase (endometrium)
1	49	1500	10	10	Proliferative
2	43	1000	9	10	Proliferative
3	50	1140	6	10	Proliferative
4	45	950	6	10	Proliferative
5	40	800	5	10	Proliferative
6	35	1300	17	6	Proliferative
7	40	1300	12	5	Proliferative
8	45	750	10	5	Proliferative
9	48	450	8	5	Proliferative
10	50	440	6	5	Proliferative
11	30	700	16	3	Proliferative
12	38	2400	16	3	Proliferative
13	48	2500	12	3	Proliferative
14	42	440	7	1	Proliferative
15	50	600	7	10	Secretory
16	50	840	6	10	Secretory
17	47	1050	11	1	Secretory
18	42	1050	8	12	Disordered

1 Size: largest diameter.

**Figure 1:**
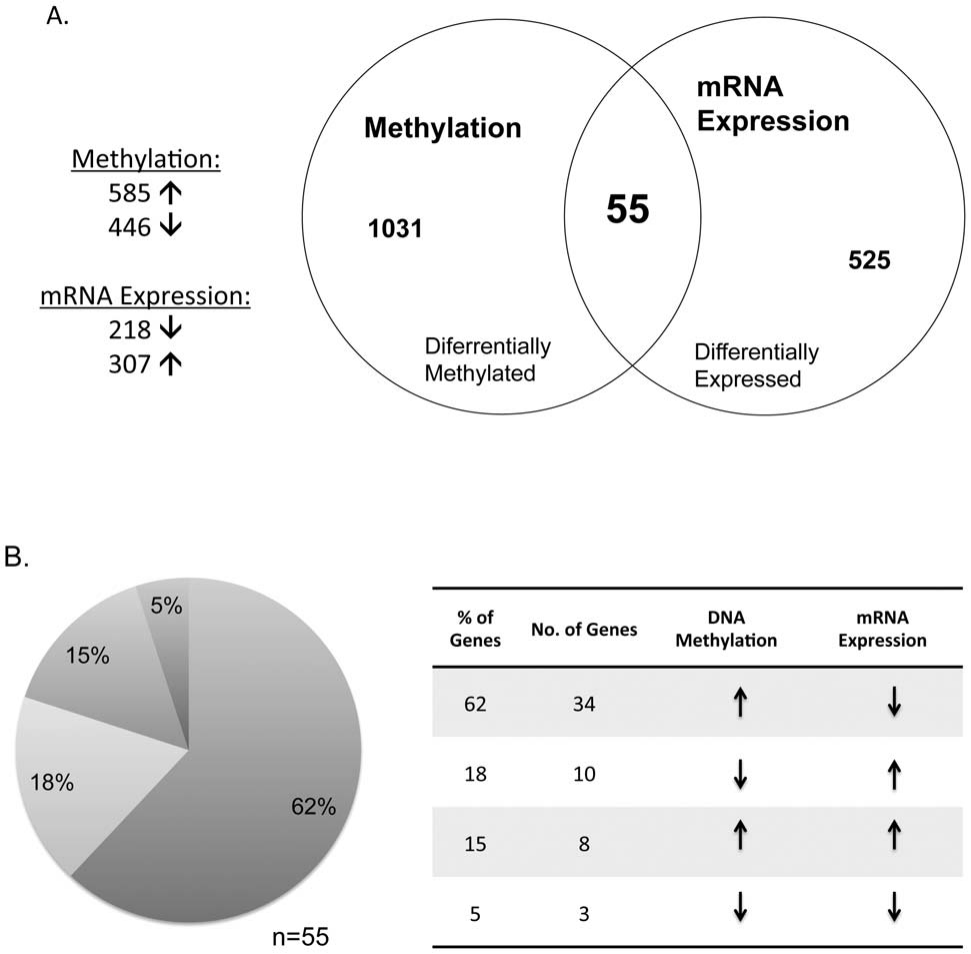
Integration of DNA methylation and mRNA expression data in uterine leiomyoma and matched myometrial tissues. (A) The Venn diagram integrates the number of differentially DNA methylated genes with the number of differentially expressed genes in leiomyomas compared to myometrial tissues. Approximately 55 genes showed changes in methylation status and expression. (B) A total of 34 (62%) of the genes were hypermethylated and downregulated, 10 (18%) were hypomethylated and upregulated, 8 (15%) were hypermethylated and upregulated and 3 (5%) were hypomethylated and downregulated.

We first compared the DNA methylation patterns in uterine leiomyoma versus adjacent normal myometrium following these thresholds: fold change >2, P<0.001, and FDR (false discovery rate)<0.01. A total of 585 transcriptional regulatory regions were hypermethylated, and 446 were hypomethylated in uterine leiomyoma compared with adjacent normal myometrial tissue (Figure 1A). We then analyzed mRNA expression patterns using the following criteria: fold change >1.5, P<0.001 and FDR<0.01. We found that 307 mRNA species were downregulated, and 218 were upregulated in uterine leiomyoma compared with myometrial tissue (Figure 1A). We then analyzed the relationship between the DNA methylation of a transcriptional regulatory region and mRNA expression of the gene in that region. A total of 55 genes showed both differential DNA methylation and changes in mRNA expression in uterine leiomyoma and adjacent normal myometrial tissue (Figure 1A). Compared with the myometrium, uterine leiomyoma contained 34/55 genes (62%) that were hypermethylated and transcriptionally downregulated and 10/55 genes (18%) that were hypomethylated and transcriptionally upregulated (Figure 1B). Thus, 44/55 genes (80%) showed an inverse correlation between promoter region methylation and mRNA expression (Table 2 and 3). We also observed that 15% of the overlapping genes were hypermethylated and transcriptionally upregulated, and a much smaller number (5%) were hypomethylated and downregulated.

**Table 2:** Summary of hypermethylated, transcriptionally downregulated genes in uterine leiomyoma compared with adjacent normal myometrium (N = 18 matched samples).

Gene symbol	Gene name	DNA Methylation^1^	P-value	mRNA level^2^	P-value
KRT19	Keratin 19	12.81	4.92E-10	−2.5166	5.68E-05
NUAK1	NUAK family, SNF1-like kinase, 1	7.327	9.98E-07	−1.5532	6.70E-06
KLF11	Kruppel-like factor 11	7.1816	1.40E-07	−1.6765	1.32E-05
DLEC1	deleted in lung and esophageal cancer 1	6.3828	1.01E-07	−1.51	1.44E-03
CORIN	Corin, serine peptidase	5.9463	2.85E-09	−1.5615	2.17E-05
EFEMP1	EGF-containing fibulin-like extracellular matrix protein 1 isoform b	5.6828	4.02E-09	−3.32	9.22E-06
MBP	Myelin basic protein	5.5791	1.83E-06	−1.7654	1.50E-04
TMEM173	Transmembrane protein 173	5.1523	7.89E-07	−1.6351	2.56E-04
TNFSF10	Tumor necrosis factor (ligand)	4.9483	1.51E-08	−1.9423	1.16E-07
BST2	Bone marrow stromal cell antigen 2	4.5596	9.82E-11	−2.1626	1.44E-06
C1orf115	Chromosome 1 open reaing frame 115	3.9093	9.45E-07	−1.7191	4.16E-08
HOXA5	Homeobox A5	3.7552	8.49E-08	−1.7155	7.35E-06
TEK	TEK tyrosine kinase, endothelial	3.7477	9.69E-09	−1.8461	1.37E-06
RBP1	Retinol binding protein 1, cellular	3.686	1.10E-08	−2.3092	1.14E-04
RASIP1	Ras interacting protein 1	3.5885	1.06E-11	−1.7365	9.28E-05
GRAMD3	GRAM domain containing 3	2.8256	1.19E-05	−1.5505	1.03E-05
CCDC109B	Coiled-coil domain containing 109B	2.645	1.60E-05	−1.5931	4.92E-05
APOLD1	Apolipoprotein L domain containing 1	2.6009	1.45E-12	−1.9577	6.72E-05
CALCRL	Calcitonin receptor-like	2.5571	2.18E-06	−1.856	3.63E-07
SERPINF1	Serpin peptidase inhibitor, clade F	2.4166	3.88E-05	−1.8676	9.71E-04
TM4SF1	Transmembrane 4 L six family	2.2707	7.66E-08	−1.9102	3.64E-07
CD34	CD34 molecule	2.2474	1.41E-09	−1.585	1.51E-05
CFB	Complement factor B	2.2336	3.08E-04	−1.9627	4.53E-06
SRGN	Serglycin	2.2307	2.10E-05	−1.8865	1.33E-06
LYVE1	Lymphatic vessel endothelial hyaluronan receptor 1	2.2275	9.19E-07	−2.4155	1.90E-05
LCN6	Lipocalin 6	2.1991	2.38E-10	−1.5541	2.34E-06
PCOLCE	Procollagen C-endopeptidase enhancer	2.1615	2.83E-05	−2.2239	1.87E-05
DARC	Duffy blood group, chemokine receptor	2.1597	1.44E-09	−3.5596	4.90E-06
CLDN5	Claudin 5	2.1438	1.44E-09	−1.6468	7.82E-05
S1PR1	Sphingosine-1-phosphate receptor 1	2.1421	1.44E-09	−1.592	2.52E-06
CCL21	Chemokine (C-C motif) ligand 21	2.1272	2.65E-07	−1.9191	5.97E-05
HTATIP2	HIV-1 Tat interactive protein 2, 30 kDa	2.1085	1.19E-04	−1.6651	1.88E-06
SOX18	SRY (sex determining region Y)-box 18	2.0969	9.81E-08	−1.699	5.79E-05
CREG1	Cellular repressor of E1A-stimulated genes 1	2.0799	4.55E-05	−1.6091	3.35E-05
PECAM1	Platelet/endothelial cell adhesion molecule	2.0659	3.79E-08	−1.6298	9.27E-06
CRIM1	Cysteine rich transmembrane BMP regulator 1	2.0092	1.84E-08	−1.6596	1.51E-05

1 Fold change was calculated as mean methylation beadchip value for leiomyoma relative to normal myometrium.

2 Fold change was calculated as mean mRNA expression microarray value for leiomyoma relative to normal myometrium.

**Table 3:** Summary of hypomethylated, transcriptionally upregulated genes in uterine leiomyoma compared with adjacent normal myometrium (N = 18 matched samples).

Gene symbol	Gene name	DNA Methylation^1^	P-value	mRNA level^2^	P-value
POPDC2	Popeye domain containing 2	−6.0818	3.73E-07	1.7758	1.14E-04
PCP4	Purkinje cell protein 4	−4.2627	3.38E-08	3.2515	2.91E-04
IL17B	Interleukin 17B	−4.0467	1.66E-08	3.0582	3.46E-06
CHRDL2	Chordin-like 2	−2.8997	3.19E-06	2.8911	1.43E-04
RPE65	Retinal pigment epithelium-specific	−2.8382	3.57E-06	1.623	6.00E-04
PHLDB2	Pleckstrin homology-like domain,	−2.6662	1.11E-10	1.5018	5.80E-05
MMP11	Matrix metallopeptidase 11	−2.585	9.82E-07	4.921	1.85E-04
MFAP2	Microfibrillar-associated protein 2	−2.2917	4.96E-06	2.1194	2.81E-06
JPH4	Junctophilin 4	−2.2868	5.50E-08	1.5724	3.39E-06
PLP1	Proteolipid protein 1	−2.1274	4.06E-04	3.3833	6.71E-05

1 Fold change was calculated as mean methylation beadchip value for leiomyoma relative to normal myometrium.

2 Fold change was calculated as mean mRNA expression microarray value for leiomyoma relative to normal myometrium.

### Patterns of differential DNA methylation and mRNA expression in uterine leiomyoma and matched adjacent myometrial tissues

**Figure 2:**
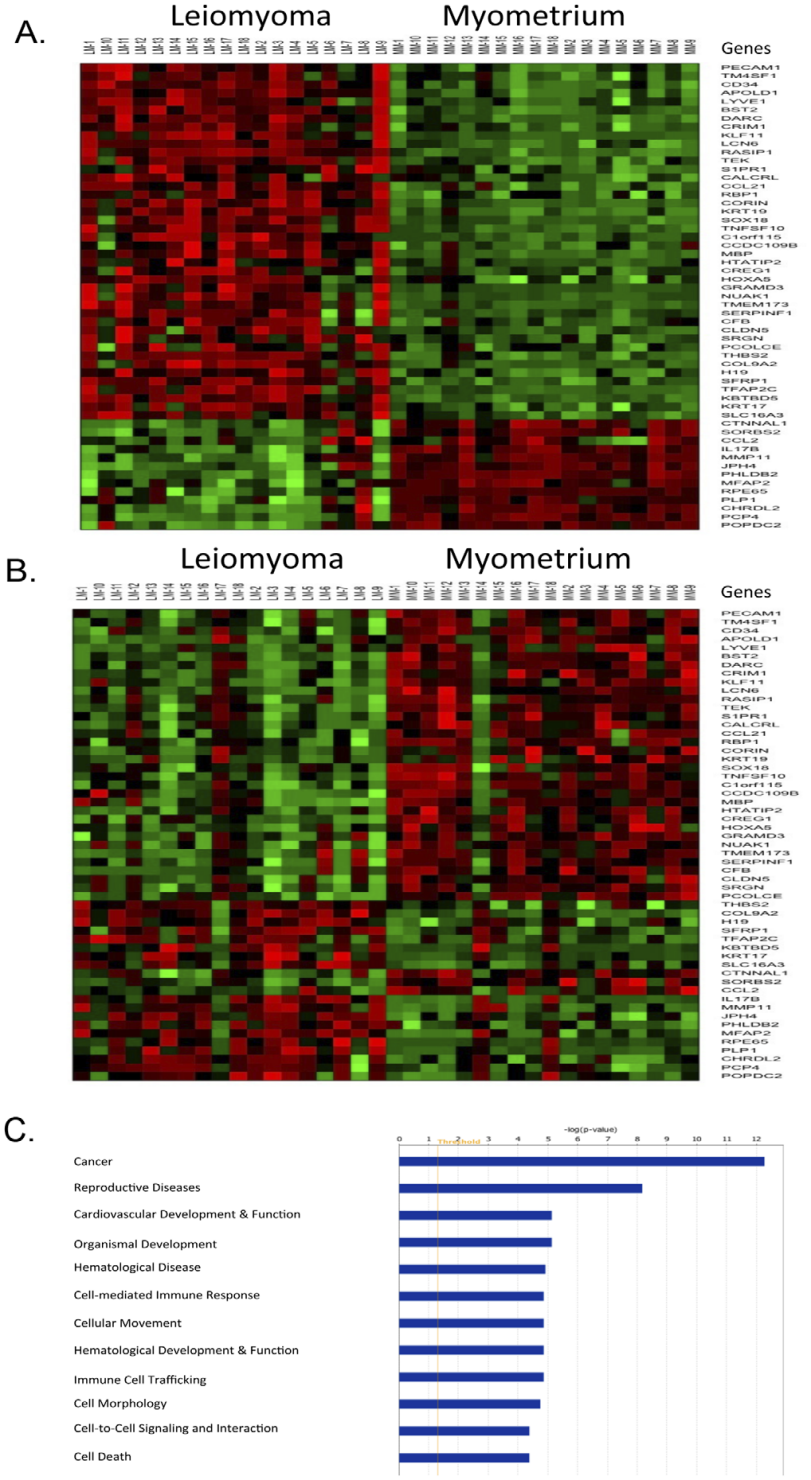
Differential patterns of DNA methylation and mRNA expression in uterine leiomyoma and matched myometrial tissues. Overall, 36 samples from 18 subjects were analyzed; 18 samples were obtained from leiomyomas and 18 from adjacent matched myometrial tissues. (A) The DNA methylation profile shows that most of the samples exhibit a very homogeneous DNA methylation pattern. (B) The gene expression profile exhibits a less homogeneous pattern, which could be due to mRNA instability. (C) Functional analysis of the 55 genes with correlation between DNA methylation status and mRNA expression showed that the top two functions represented are cancer processes (P<10^−12^) and reproductive system disease (P<10^−8^).

We further analyzed the group of 55 genes that overlapped with respect to differential DNA methylation and mRNA expression. The majority of the 18 uterine leiomyoma samples exhibited a homogeneous pattern of DNA hypermethylation, whereas the normal myometrial samples were largely hypomethylated (Figure 2A). Intriguingly, while differential mRNA expression in the uterine leiomyoma and adjacent normal myometrial samples exhibited a more heterogeneous pattern (Figure 2B), the pattern was a mirror image of the differential DNA methylation pattern (Figure 2A). We also performed a functional analysis of the 55 overlapping genes using Ingenuity Pathways Analysis (IPA) and the Bioconductor GeneAnswers package, and found that based on their p-values level, the top two most significantly enriched gene functions are cancer processes (P<10^−12^) or reproductive system diseases (P<10^−8^) [13]. The genes involved in cancer were DLEC1, KRT19, KLF11, SERPINF1, TEK, APOLD1, LYVE1, CCL2, IL17B, and TNFS10, and genes involved in reproductive system diseases were CRIM1, PCP4, CHRDL2, HOXA5, PLP1, COL9A2, SOX18, BMP, CALCRL, SFRP1 (Figure 2C).

### Validation of differential DNA methylation using bisulfite genomic sequencing

We hypothesized that the 55 overlapping genes with differential DNA methylation and mRNA expression in uterine leiomyoma compared with normal myometrium were likely to be true targets of epigenetic regulation in uterine leiomyoma. Initially, we examined the regulatory CpG islands in the promoter regions of selected genes from the 55 candidates, and characterized the positions of 5′ CpG islands and transcriptional start sites using available genome databases. From this set, we then selected three of the hypermethylated genes, Kruppel-like transcription factor 11 (KLF11), deleted in lung and esophageal cancer 1 (DLEC1), keratin 19 (KRT19) for further analysis based on their known tumor suppressor functions.

**Figure 3:**
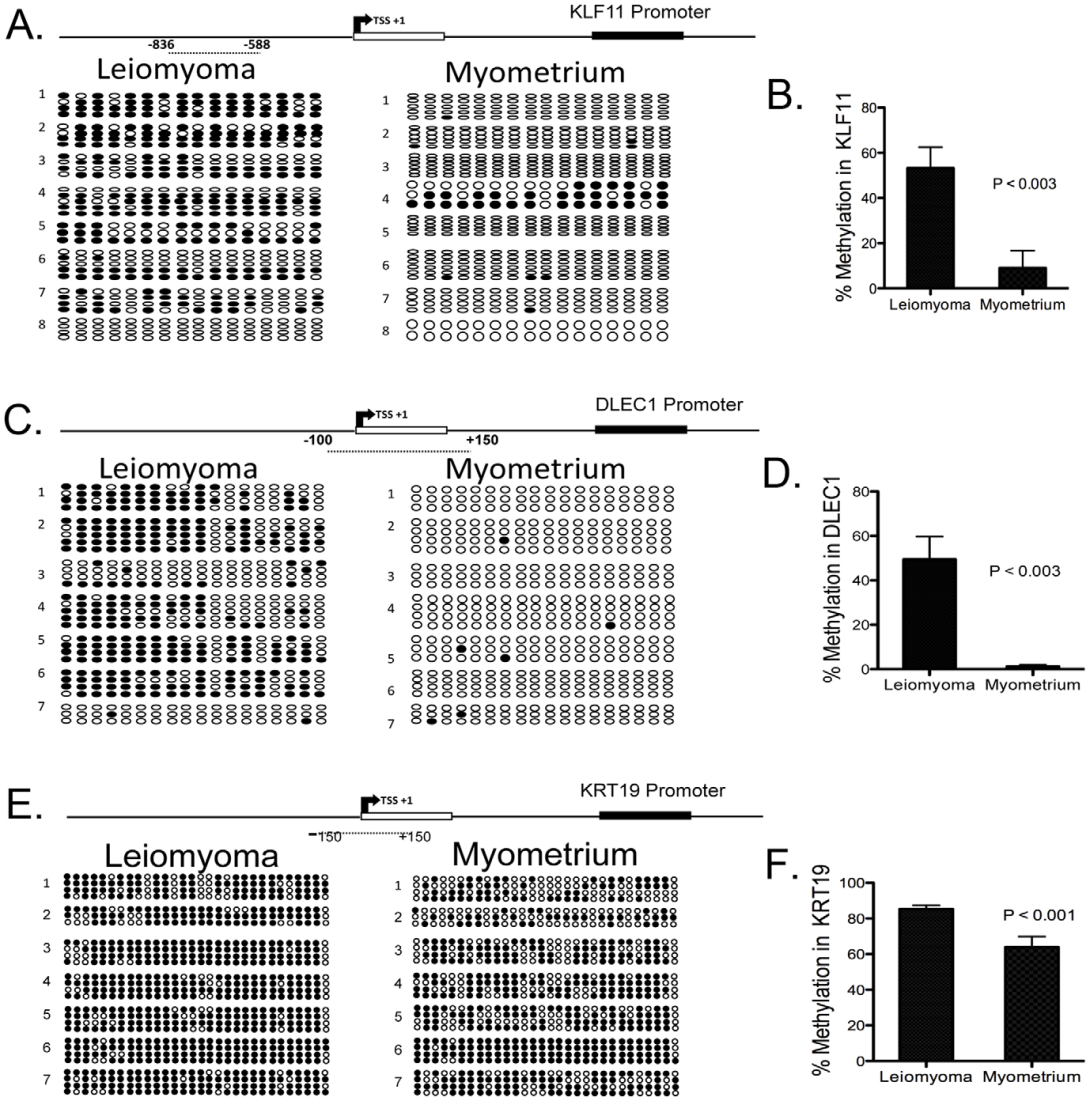
DNA methylation status of KLF11, DLEC1, and KRT19 promoters in uterine leiomyoma and matched myometrial tissues. (A) DNA methylation status of 16 CpG sites in the KLF11 promoter region obtained from bisulfite sequencing of uterine leiomyoma and matched myometrial tissues. The numbers 1 to 8 on the side represent subjects from whom tissues were obtained, (#1–4) subjects were African Americans included in our original genome-wide DNA methylation study and we incorporated 4 new matched samples from Caucasian subjects (#5–8). Black circles represent methylated cytosines and white represent unmethylated cytosines. Numbers indicate the position of cytosine residues of CpGs relative to the transcription start site (+1). (B) Percent DNA methylation of the KLF11 promoter region in uterine leiomyoma and myometrial tissues, P<0.003. (C) DNA methylation status of 18 CpG sites in the DLEC1 promoter region from 3 African American (#1–3) and 4 Caucasian subjects (#4–7). (D) Percent DNA methylation of the DLEC1 promoter region in uterine leiomyoma and myometrial tissues, P<0.003. (E) DNA methylation status of 30 CpG sites in the KRT19 promoter region from 4 African American (#1–4) and 3 Caucasian subjects (#4–7). (F) Percent DNA methylation of the KRT19 promoter region in uterine leiomyoma and myometrial tissues, P<0.001.

First, we studied the KLF11 promoter via sequencing of bisulfite-treated genomic DNA from uterine leiomyoma and myometrial tissues from 8 subjects. Four of these were African American that were included in our original genome-wide DNA methylation study (#1–4), and we incorporated four new matched samples from Caucasian subjects (# 5–8). We analyzed the DNA methylation status of a cluster of 16 CpG dinucleotides across a 249-bp region of a CpG island, located approximately −900 bp to −500 bp upstream of the KLF11 promoter region (Figure 3A). Four to six clones were sequenced from each subject. The detailed CpG methylation level of primary leiomyoma (n = 8) and matched myometrial (n = 8) tissues verified the hypermethylated state of the KLF11 promoter in uterine leiomyoma compared with adjacent normal myometrium. Six of the eight uterine leiomyoma samples showed increased DNA methylation of the KLF11 promoter. In uterine leiomyoma, the majority of the 16 CpG dinucleotides in the KLF11 promoter were consistently methylated. There was a significant statistical difference (P<0.004, Student's t-test) in DNA methylation levels between the uterine leiomyoma and matched myometrial tissues (Figure 3B).

Then, we analyzed the promoter region of another tumor suppressor gene, DLEC1, in uterine leiomyoma and myometrial samples from 7 subjects. Three subjects were African Americans included in our original genome-wide DNA methylation study (#1–3), and we incorporated four new matched samples from Caucasian subjects (#4–7). We sequenced a cluster of 18 CpG dinucleotides across a 252-bp region of a CpG island located within a −100 to +150 bp region of the DLEC1 promoter (Figure 3C). Uterine leiomyoma tissues demonstrated a dense methylation pattern at the DLEC1 promoter region in 5 of the 7 subjects. The majority of the 18 CpG dinucleotides in the DLEC1 promoter were consistently methylated in uterine leiomyoma, but not in normal myometrial tissues. Overall, there was a significant statistical difference (P<0.003, Student's t-test) in methylation levels between uterine leiomyoma and matched normal myometrial tissues (Figure 3D).

Finally, we studied the KRT19 promoter via sequencing of bisulfite-treated genomic DNA from uterine leiomyoma and myometrial tissues from 7 subjects. Four subjects were African Americans included in our original genome-wide DNA methylation study (#1–4), and we incorporated three new matched samples from Caucasian subjects (#5–7). We analyzed the DNA methylation status of a cluster of 30 CpG dinucleotides across a 300-bp region of a CpG island, located approximately −150 bp to −150 bp around the KRT19 promoter (Figure 3F). Four to six clones were sequenced from each subject. The detailed CpG methylation level of primary leiomyoma (n = 7) and matched myometrial (n = 7) tissues verified the hypermethylated state of the KRT19 promoter in uterine leiomyoma compared with adjacent normal myometrium. All seven analyzed samples showed increased DNA methylation of the KRT19 promoter. In uterine leiomyoma, the majority of the 30 CpG dinucleotides in the KRT19 promoter were consistently methylated. There was a significant statistical difference (P<0.001, Student's t-test) in DNA methylation levels between the uterine leiomyoma and matched myometrial tissues (Figure 3F). The Illumina platform covers 50 bp regions, whereas bisulfite sequencing evaluates a 250–300 bp region, which overlaps with the 50 bp sequence of interest. These longer fragments enhance fidelity.

### Impact of DNA methylation on gene expression in human uterine leiomyoma and matched adjacent myometrial tissues

**Figure 4:**
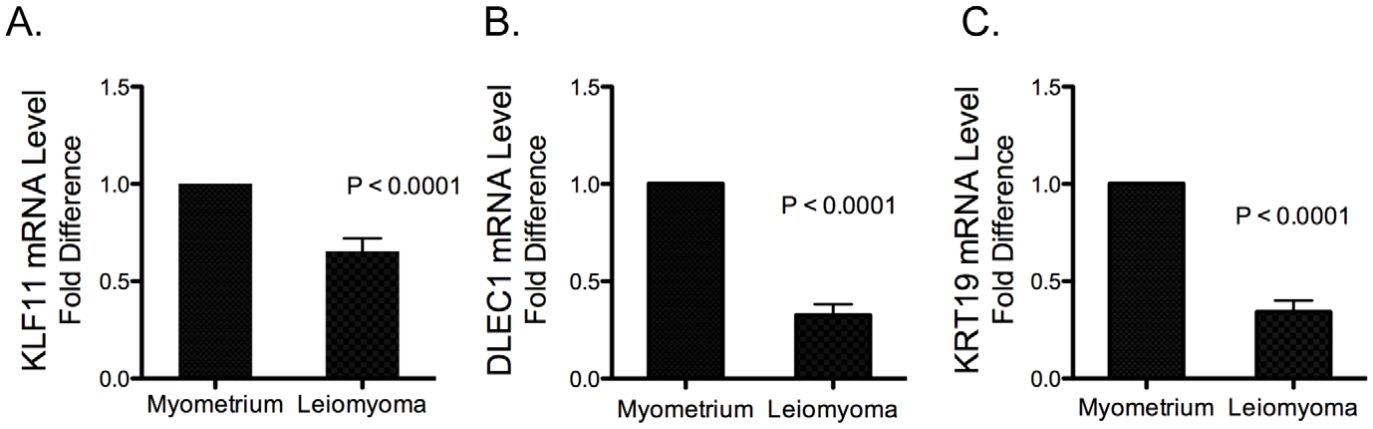
mRNA levels of KLF11, DLEC1 and KRT19 in uterine leiomyoma and matched adjacent myometrial tissues. mRNA levels of (A) KLF11, (B) DLEC1, and (C) KRT19 were quantified using all 18 samples used in microarrays plus the addition of 7–10 new Caucasian samples. mRNA levels in leiomyoma and matched myometrium were quantified by real-time PCR, they were first normalized to GAPDH. Then, to allow comparisons of data obtained from samples from different subjects, mRNA levels in the myometrial tissues were normalized to 1. KLF11 (n = 25; P<0.0001), DLEC1 (n = 28; P<0.0001) and KRT19 (n = 25; P<0.0001). The data are shown as the mean +/− SEM. P<0.05 versus myometrium tissues.

To validate that DNA methylation leads to gene silencing of the tumor suppressor genes KLF11, DLEC1, and KRT19, we assessed mRNA levels *in vivo* using real-time RT-PCR in uterine leiomyoma and matched myometrial tissues. We performed real-time RT-PCR on all 18 samples originally used in the microarray analysis plus we incorporated 7–10 new samples from Caucasian subjects. KLF11 mRNA levels in uterine leiomyoma (18 samples from original microarrays and 7 new Caucasian samples) were considerably lower (40%) than those in matched adjacent normal myometrial tissues (n = 25; P<0.0001, Figure 4A). DLEC1 mRNA levels in uterine leiomyoma tissues (18 samples from original microarray and 10 new Caucasian samples) were also significantly lower (60%) than those in matched myometrial tissues (n = 28; P<0.0001, Figure 4B). KRT19 mRNA levels in uterine leiomyoma (18 samples from original microarray and 7 new Caucasian samples) were considerably lower (66%) than those in matched adjacent normal myometrial tissues (n = 25; P<0.0001, Figure 4C). We have not observed any differences in mRNA levels between samples from African- and Caucasian-American subjects.

### The effects of chemical demethylation of CpG dinucleotides on mRNA levels

**Figure 5:**
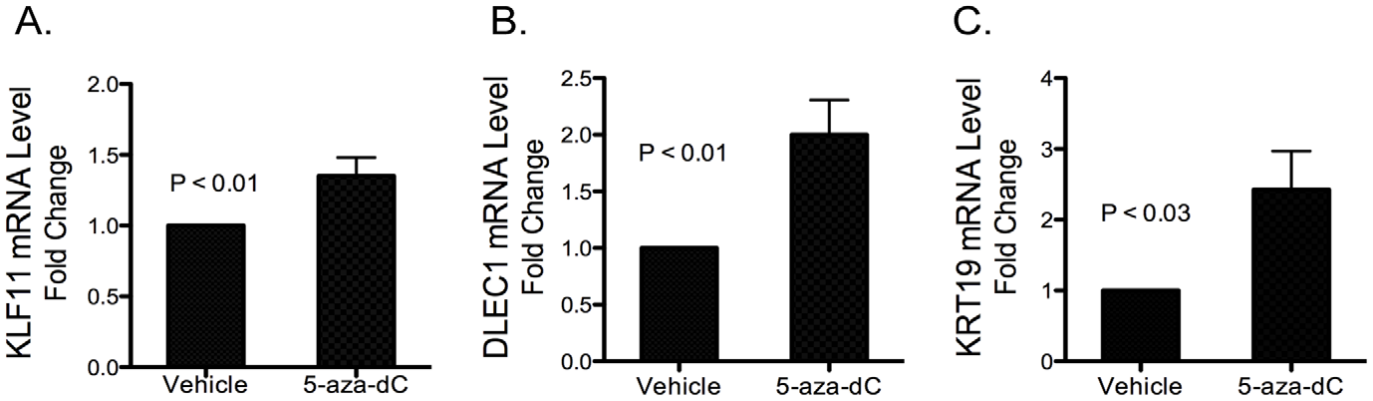
Effect of DNMT inhibitor 5-aza-dC on KLF11, DLEC1 and KRT19 expression in leiomyoma smooth muscle cells. (A) KLF11, (B) DLEC1 and (C) KRT19 mRNA levels were quantified by real-time RT-PCR after treatment of cultured uterine leiomyoma smooth muscle cells with vehicle or 5-aza-dC (3 µM) for 5 days. mRNA levels were normalized first to GAPDH and then, to those in vehicle-treated cells. Experiments were performed in triplicate samples, and the data are representative of samples from seven different subjects. The data are shown as the mean +/− SEM. P<0.05 as compared with vehicle treatment.

To determine whether the decrease in mRNA expression of these three tumor suppressor genes is regulated by DNA methylation, primary cultured leiomyoma smooth muscle cells isolated from 7 new subjects not previously used in microarrays (4 subjects were African- and 3 Caucasian-American) were treated with the DNMT inhibitor, 5-aza-dC at different concentrations (0.5, 1, 3, 5, 10, 15, 20 µM) and time points (1, 3, 5 days). Real-time RT-PCR was performed to measure KLF11, DLEC1, and KRT19 mRNA levels. We observed that 5-aza-dC treatments at various doses had a similar effect on restoring mRNA levels. We chose the 3 µM dose to perform the subsequent experiments because it was potentially less toxic to the cells while being maximally effective. After analyzing mRNA expression after 1, 3 and 5 days, we determined that the effect is most effective at restoring mRNA expression levels after 5 days of treatment. As shown in Figure 5, 5-aza-dC treatment of primary cultured leiomyoma smooth muscle cells led to an increase in KLF11 mRNA by 1.4-fold, DLEC1 mRNA by 2-fold, and KRT19 mRNA by 2.4-fold suggesting that these three genes are epigenetically regulated in leiomyomas.

### Protein expression in human uterine leiomyoma and matched adjacent myometrial tissues

**Figure 6:**
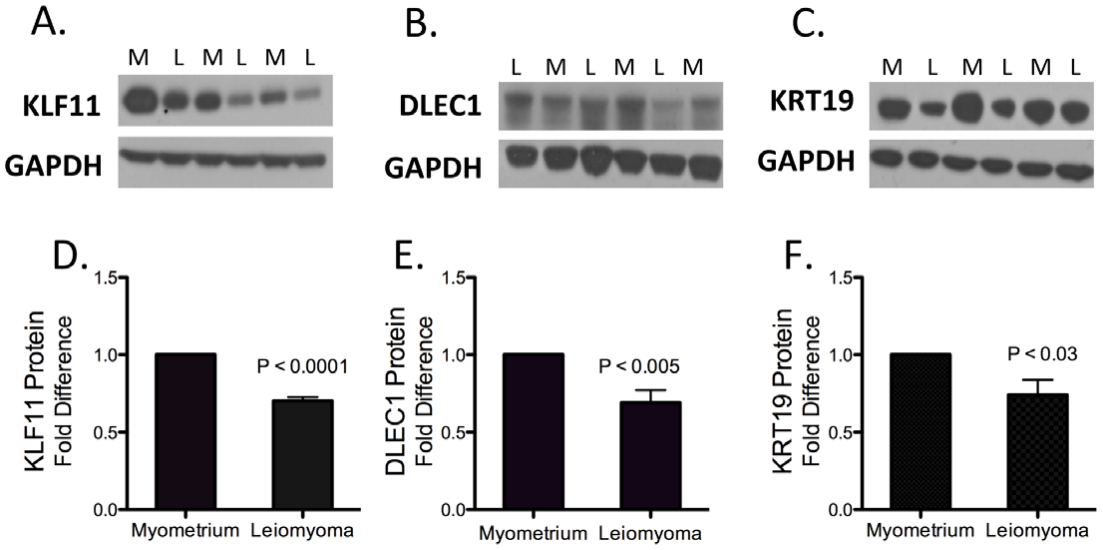
Protein levels of KLF11, DLEC1 and KRT19 in uterine leiomyoma and matched adjacent myometrial tissues. Protein levels of KLF11, DLEC1 and KRT19 in leiomyoma and matched myometrium were analyzed by immunoblotting. (A–C) Western blots are representative of several experiments repeated in at least 6 subjects. (D–F) Protein quantification using ImageJ software shows that there is lower expression of KLF11 (n = 6, P<0.0001), DLEC1 (n = 9, P<0.005) and KRT19 (n = 8, P<0.03) (Student's t-test) in leiomyoma compared to matched adjacent myometrial tissues. GAPDH was used as a loading control. M = myometrial tissue; L = leiomyoma tissue.

To understand the *in vivo* relevance of how DNA methylation affects gene function, we analyzed protein levels of KLF11, DLEC1 and KRT19 in human leiomyoma and matched normal myometrial tissues using western blot. KLF11 protein levels in all 6 subjects were significantly lower (30%) in leiomyoma compared with myometrial tissues (Figure 6A and D). Overall, DLEC1 protein levels were also significantly lower (30%) in leiomyoma than in myometrial tissues, and only 2 out of 9 subjects had no difference in DLEC1 expression in leiomyoma compared with myometrial tissues (Figure 6B and E). KRT19 protein levels in 8 subjects were lower (25%) in leiomyoma than myometrial tissues, and only 1 subject had higher KRT19 protein levels in leiomyoma compared with myometrial tissues (Figure 6C and F). All protein studies were performed with 6–9 new pairs of matched samples not previously used in the microarray experiments; 5 subjects were African American and 4 subjects were Caucasian. Overall, western blots showed that KLF11 (n = 6, P<0.0001), DLEC1 (n = 9, P<0.005) and KRT19 (n = 8, P<0.03) (Student's t-test). Protein levels in leiomyoma tissues were significantly lower compared with matched normal myometrial tissues.

## Discussion

Recent evidence suggests that DNA is differentially methylated in uterine leiomyoma versus adjacent normal myometrial tissue; however, these findings are predominantly reported in small studies and analysis of individual candidate genes such as ESR1, which has been shown to be hypomethylated in leiomyomas [9]–[10]. We particularly paid attention to the ESR1 gene, but we did not observe any differential DNA methylation patterns between leiomyoma and myometrium. Hypomethylation of ESR1 in leiomyoma was reported using a group of Japanese subjects; thus the difference between our findings and theirs could be attributed to racial differences. Similar racial differences have also been reported for the aromatase mRNA levels and promoter usage in uterine leiomyomas [14].

More recently published reports have attempted to demonstrate differential DNA methylation in leiomyomas; one study examined differences across the X chromosome in a single subject supporting the concept of epigenetic regulation in uterine leiomyoma. However, the other study was insufficient to identify differences in DNA methylation, which could be due to the small number of samples investigated [15]–[16]. Here, we report the first genome-wide analysis of differential DNA-methylation and mRNA expression in uterine leiomyoma and adjacent normal myometrial tissues from 18 matched pairs, all from African American subjects to limit biological heterogeneity, and avoid epigenetic variations among ethnic groups. The following real-time RT-PCR validation of mRNA expression and bisulfite sequencing validation of DNA methylation of these genes were performed on both a subset of the original African American samples and additional new samples from Caucasian subjects. Additionally, *in vitro* cultured experiments utilized primary cells from both ethnic groups. We have not observed any apparent differences with respect to the 3 studied genes between samples from African- and Caucasian-American subjects suggesting that the findings may be applicable to both ethnic groups. This conclusion, however, should be taken with some caution due to the low number of Caucasian subjects. Further studies are needed to make a more definitive conclusion.

Our study confirms the link between epigenetic DNA modifications and gene expression in uterine leiomyomas, by demonstrating the effects of promoter DNA methylation on gene silencing, particularly in three tumor suppressors known to be involved in reproductive tumorigenesis. Though our work is the first to examine genome-wide analysis of DNA methylation in uterine leiomyoma and there are no existing data against which we can compare our results, our mRNA expression profiles are consistent with previously published reports [17]–[26].

In our genome-wide analysis, we observed that 1,031 transcriptional regulatory regions were differentially methylated and only 525 mRNA species were transcriptionally altered in uterine leiomyoma compared with myometrial tissue. This degree of mismatch between DNA methylation and steady-state mRNA levels was expected since changes in DNA methylation may not always lead to changes in steady-state mRNA levels for the following potential reasons. (i) DNA methylation alone may not be sufficient to alter mRNA expression, and other events such as changes in the structure of chromatin formed on a methylated template are needed to render it transcriptionally altered. (ii) The availability and binding capacity of specific transcription factors are needed to regulate the rate of mRNA transcription from a gene promoter. (iii) Finally, other factors that regulate the half-life of a certain transcript will determine its steady-state levels. Consequently, it is expected that changes in steady-state mRNA levels are regulated only partially by DNA methylation.

Although, we attempted to account for differences in the menstrual cycle, the majority of the samples included in our analysis (14/18) were obtained during the proliferative phase. Moreover, it is challenging to date the endometrium for cycle phase since many women with uterine leiomyomas have irregular cycles with prolonged bleeding. The small number of secretory phase samples did not permit us to compare biological differences as a function of the cycle phase. Since the correlation between differences in DNA methylation and gene expression was evaluated in paired samples from the same patient, the effect of cycle phase on this analysis was further minimized.

In this study, we noted a key epigenetic mechanism whereby increased promoter methylation leads to transcriptional suppression in uterine leiomyoma compared with matched normal myometrial tissues. The second predominant mechanism was hypomethylation associated with overexpression of genes indicating an overall inverse relationship between DNA methylation and gene expression in uterine leiomyoma. However, we also observed some genes to be hypermethylated and upregulated, and other genes to be hypomethylated and downregulated. The absence of an inverse relationship between promoter DNA methylation and mRNA expression in this minor group of genes is consistent with previously published data. For example, methylation of one particular CpG island in the NR5A1 gene is associated with transcriptional suppression, whereas methylation of another CpG island located 4 kb downstream is associated with overexpression of NR5A1 mRNA [17], [27]. It is conceivable that the effects of a single methylated CpG island on gene expression may be either gene-specific or location-specific within the same gene.

We verified the effects of promoter DNA methylation on transcriptional inhibition of three tumor suppressor genes namely, KLF11, DLEC1, and KRT19. KLF11 is a transcription factor and a member of the transforming growth factor beta (TGFβ) family, which is involved in key cellular functions such as apoptosis, proliferation, and differentiation [28]. KLF11 is expressed in a number of human tissues, and it is repressed in several human cancers. It inhibits neoplastic transformation and cell growth both *in vivo and in vitro* [29]. We previously demonstrated the downregulation of KLF11 expression in uterine leiomyoma tissues compared with normal matched myometrial tissue [30]. Although the mechanism involved in KLF11-regulated cell proliferation is not fully understood, we demonstrated for the first time that KLF11 is epigenetically regulated by DNA methylation, with hypermethylation correlating with a repressed state in uterine leiomyoma. Recently, KLF11 was also shown to be aberrantly hypermethylated in myelodysplastic syndromes. It has been suggested that KLF11 inhibits gene expression through a Sin3a-HDAC interacting domain and recruitment of the corepressor mSin3a [31]. We plan to investigate this mechanism further, and identify the DNMTs and DNA methyl binding proteins that are involved in silencing of KLF11.

DLEC1 is an epigenetically modified tumor suppressor gene [32]. DLEC1 is localized in the cytoplasm ubiquitously expressed in all human tissues, and repressed in several human cancers. Hypermethylation of the DLEC1 promoter is associated with its transcriptional repression in a wide variety of malignant tumors originating from lung, esophagus, kidney, ovary, nasopharynx, and liver [33]. The DLEC1 promoter region contains a CpG island in the first exon, and we demonstrated here that methylation of this CpG is responsible for the repression of DLEC1 expression in uterine leiomyoma. Our analysis revealed a strong association between silencing of DLEC1 expression and promoter hypermethylation in uterine leiomyoma; in addition, treatment of addition of cultured primary uterine leiomyoma smooth muscle cells with a DNMT inhibitor restored DLEC1 expression. The DLEC1 gene encodes a 166 kDa protein, whose biologic function remains unknown due to lack of homology to any known conserved proteins or domains [34]. In the future, we plan to characterize the biological function of DLEC1 in uterine leiomyoma.

KRT19 is an intermediate filament protein responsible for the structural integrity of epithelial cells, this genes encodes a 40-kDa protein [35]. In mammalian cells, keratin filaments are organized in a complex network spreading from the nucleus to the cytoplasmic membrane. KRT19 is also known as an epigenetically regulated tumor suppressor gene, which has frequently demonstrated promoter hypermethylation associated with transcriptional downregulation in several cancerous tumors such as neuroblastomas, squamous cell carcinoma of the head and neck region and renal cell carcinomas [36]–[38]. Also, it is one of the most common used markers for real-time RT-PCR detection of tumor cells disseminated in lymph nodes, peripheral blood and bone marrow of breast cancer patients [39]–[40].

Using genome-wide analyses of DNA methylation in uterine leiomyoma we hope to define a specific epigenetic profile that could inform the development of diagnostic biomarkers for uterine leiomyoma as well as identify potential therapeutic targets. Because DNA methylation is reversible, epigenetic modifying drugs could be used in the medical management of uterine leiomyoma. Importantly, aberrant DNA methylation and other epigenetic abnormalities may represent a critical initial mechanism that triggers transformation of a single myometrial cell that will eventually give rise to a monoclonal leiomyoma tumor. Understanding the mechanism underlying the pathogenesis of uterine leiomyoma will be critical for developing new preventive and therapeutic approaches to the disease.

## Materials and Methods

### Ethics Statement

To obtain human tissues, we followed the protocol approved by the Institutional Review Board for Human Research of Northwestern University and New York University. Written informed consent was received from all subjects.

### Tissue acquisition

For *in vivo* studies, we obtained matched pairs of leiomyoma and adjacent myometrium from a total of 23 African American and 14 Caucasian-American subjects undergoing hysterectomy for symptomatic fibroids. To minimize heterogeneity due to race we used samples from 18 African American subjects for both genome-wide DNA methylation and gene expression microarrays. In follow-up verification studies, we included samples from 4 of the original African American group plus 4 additional Caucasian subjects for bisulfite sequencing and all 18 original African American plus 10 Caucasian subjects for mRNA quantification using real-time RT-PCR. Samples from Caucasian subjects were added to evaluate whether similar patterns of DNA methylation and mRNA expression were observed.

Key clinical characteristics of the 18 African American subjects, whose samples were used for both microarrays are described in Table 1. The clinical characteristics of Caucasian subjects fall within the range depicted in Table 1. All African- or Caucasian-American subjects were premenopausal women (mean age 44 years; range 30–50 years), and the tumor sizes ranged from 5 to 17 cm in diameter. The subjects had not received any hormonal treatment for at least 6 months prior to surgery. The cycle phase was estimated by the last menstrual period and was confirmed by endometrial histology. Each leiomyoma tissue biopsy was obtained at 1 cm from the outer capsule of the tumor. The matched myometrial sample was taken at 2 cm from the tumor in the fundal portion of the uterus. All tissue samples were snap-frozen in liquid nitrogen before DNA and RNA isolation. The leiomyoma and myometrial tissue specimens were biopsied by a pathologist under the supervision of Dr. Wei, who is a board-certified pathologist. Dr. Wei and his pathology team carefully examined these specimens both grossly and histologically and made sure that they were not contaminated by endometrial stroma or epithelium.

The initial sample size of 18 pairs for microarray platforms was derived from the power analysis based on published clinical studies, which showed that significant differences for most dysregulated gene products can be detected with sample size of 13 to 22 subjects per group with 80% power and 0.05 alpha levels [41].

### Primary cell isolation

Leiomyoma smooth muscle cells were isolated from the peripheral portions approximately 1 cm from the outer capsule of the leiomyoma, and then cultured as previously described with minor modifications [42]. Cells were cultured in DMEM/F12 1∶1 (GIBCO/BRL, Grand Island, NY) containing 10% fetal bovine serum (Invitrogen, Carlsbad, CA) and grown in a humidified atmosphere with 5% CO2 at 37°C. Primary cells were used only up to the second passage to avoid changes in phenotype and gene expression.

### DNA methylation and mRNA expression analysis

Genomic DNA was isolated from 20 mg frozen tissues using the DNeasy Blood & Tissue (Qiagen, Valencia, CA). One microgram of genomic DNA from each sample was bisulfite-modified using the EZ DNA Methylation kit (Zymo Research, Orange, CA), according to the manufacturer's protocol along with the technical validation of the assay [12]. Bisulfite-modified DNA was hybridized to the HumanMethylation27 Beadchip (Illumina Inc., San Diego, CA).

Total RNA was isolated from 20 mg of frozen tissues using the RNeasy Fibrous Tissue kit (Qiagen) according to manufacturer protocols with minor modifications. After elution, RNA samples were quantified using a ND-1000 spectrophotometer (NanoDrop Wilmington, DE) and evaluated for degradation using a 2100 Bioanalyzer (Agilent Technologies, Santa Clara, CA). For use in hybridization, samples were required to have a RIN>9, an OD_260/280_ of 1.9–2.0, and OD_260/230_>1.5, and a 28S∶18S ribosomal band ratio of >1.5. The samples were hybridized into the HumanHT-12 v3 genome-wide gene expression BeadChips according to the manufacturer's protocol (Illumina, Inc.).

We used the Bioconductor lumi [43] package, which was developed by our collaborator and is widely used as one of the standard tools to process both Illumina DNA methylation and mRNA expression data. The data first went through a QA/QC step. For Illumina expression data, the data passing QA step was preprocessed using a variance stabilization transformation method [44] followed by quantile normalization. For methylation data, we first performed a color balance adjustment of methylated and unmethylated probe intensities between two color channels using a smooth quantile normalization method. The methylated and unmethylated probe intensities were then normalized using the SSN (Scale and Shift Normalization) method. The methylation M-value (log 2 ratio of methylated and unmethylated probes) was calculated to estimate the methylation level of the measured CpG sites [45]. The follow-up analysis was then based on the M-value. We used a shift and scaling normalization (SSN) method, which includes global background shift during normalization instead of more aggresive quantile normalization as described in reference 45. We made this decision primarily because we produced high quality and consistent data evident by the principal component analysis that we are now incorporating in the supplemental section.

After preprocessing, the differential analysis of methylation data was similar to that used for expression microarray data. Probes (for expression data) or CpG-sites (for methylation data) with all samples “Absent” (lower or around background levels) were removed from further analysis to reduce false positives. To compare the differences in both methylation and expression between leiomyoma and myometrial tissues, we performed differential analyses using routines implemented in the limma package [46]. To ensure both high statistical significance and strong biological effects, we require that the differentially methylated CpG sites had an FDR<0.01 and fold-change (based on M-value) of >2; using this process 1031 CpG sites (585 up, 446 down) were identified. For mRNA expression data, we required that the differentially expressed genes had an FDR<0.01 and a fold-change of>1.5; with these parameters, we identified 525 genes (218 up, 307 down). We mapped the differentially methylated CpG sites to the closest downstream gene, and found there are 55 overlapping genes between the lists of genes with changes in DNA methylation and mRNA expression data. The microarray data is MIAME compliant and is available at the Gene Expression Omnibus Web site (http://www.ncbi.nlm.nih.gov/geo) under accession No.GSE31699.

### Bisulfite genomic sequencing

To confirm DNA methylation levels by bisulfite sequencing, 500 ng of gDNA was treated with sodium bisulfite according to the manufacturer's instructions (Zymo Research, Orange, CA). For PCR amplification, 3 µl of bisulfite-treated DNA was added to a final volume of 20 µl. ZymoTaq PreMix (Zymo Research, Orange, CA) was used for all PCR reactions. The thermal cycler conditions were as follows: 95°C for 10 min then 40 cycles of denaturation at 95°C for 30 sec, annealing at 50°C for 2 min, and elongation at 72°C for 2 min, followed by an extension at 72°C for 7 min. PCR products were gel purified and cloned into the PCR 2.1 vector (Invitrogen, Carlsbad, CA). After transformation, 10 clones were sequenced on the Applied Biosystems 377 instrument. Methylation sites were visualized and quality control was performed using the QUMA software (http://quma.cdb/riken.jp/) and Biq analyzer.

### Real-time quantitative RT-PCR

Total RNA from fresh tissues and leiomyoma smooth muscle cells was extracted using Tri-reagent (Sigma-Aldrich, St. Louis, MO) and the RNeasy Fibrous Tissue kit (Qiagen). cDNA was prepared with qScript cDNA Supermix (Quanta BioSciences, Inc., Gaithersburg, MD) from 2 µg of RNA. Primers against KLF11 and DLEC1 and the constitutively expressed glyceraldehyde-3-phosphate dehydrogenase (GAPDH) were used as described in previous reports. Primer specificity was confirmed by the demonstration of single peaks using dissociation curves after amplification of cDNA and a lack of amplification of genomic DNA. Real-time PCR was performed to determine the relative amounts of each transcript using the DNA-binding dye SYBR green (Applied Biosystems, Foster City, CA) and the ABI Prism 7900HT Detection System (Applied Biosystems). Cycling conditions started at 50 C for 2 min, followed by 95°C for 10 min, then 40 cycles of 95°C for 15 sec and 60°C for 1 min. The cycle threshold (Ct) was placed at a set level where the exponential increase in PCR amplification was approximately parallel between all samples. Relative fold change was calculated by comparing Ct values between the target gene and GAPDH as the reference guide. The primer sequences used were: KLF11: forward: 5′-CACGATGCACACGCCGGACT-3′; reverse: 5′-TCGCTGTCATGCCGCTTCCT-3′; DLEC1: forward: 5′-GACGAAGTGAGCGCAAGC-3′; reverse: 5′-ATCCAGCCGCTGCTTATAGA-3′; GAPDH: forward: 5′-GAAGGTGAAGGTCGGAGTC-3′; reverse: 5′-GAAGATGGTGATGGGATTTC-3′; The ΔΔCt method was used to analyze the relative changes in gene expression.

### 5-aza-2′-deoxycytidine (5-aza-dC) treatment

Monolayer cultures at approximately 40% confluence were starved in serum-free medium overnight and treated with vehicle (DMSO 1∶1000) or 0.5, 1, 3, 5, 10, 15, or 20 µM of the DNMT inhibitor 5-aza-dC (Sigma-Aldrich) for 5 days. The medium was changed every 24 hrs. Total RNA was isolated using Tri-reagent (Sigma-Aldrich). All of the experiments were repeated in triplicate using samples from at least 7 new different subjects not previously used in microarrays, 4 subjects were African- and 3 Caucasian-American.

### Protein Analysis

Protein was extracted from 50 mg of frozen tissues using mammalian protein extraction reagent (Pierce, Rockford, IL). Lysates were cleared by centrifugation at 14, 000 rpm for 10 min. Equal amounts of protein (30 ug) were resolved on 4–12% Ready Gel Precast Gels (Bio-Rad Laboratories, Hercules, CA), and transferred onto PVDF membranes. The membranes were bloted with antihuman KLF11 antibodies 1∶1000 (Cell Signaling), DLEC1 1∶500 (Sigma-aldrich), and KRT19 1∶1000 (Cell Signaling). Anti-GAPDH antibody was used as a loading control. Dectection was detected using a Supersignal West Femto (Pierce). Quantification of the immunoblots was done using ImageJ software and normalized to GAPDH.

### Statistical analysis

Statistical significance was determined by Student's t test and one-way ANOVA followed by Fisher's protected least significant difference test. Significance was accepted at P<0.05.
